# Health professionals prepared for the future. Why Social Sciences and Humanities teaching in Medical Faculties matter

**DOI:** 10.15694/mep.2018.0000195.1

**Published:** 2018-09-05

**Authors:** Nicolas Lechopier, Gilles Moutot, Céline Lefève, Maria Teixeira, Roberto Poma, Guillaume Grandazzi, Anne Rasmussen

**Affiliations:** 1 Université de Lyon / EA 4148 S2HEP; 2 Université de Montpellier; 3 Université Sorbonne Paris Cité/CNRS UMR 7219 SPHERE; 4 Université Paris Diderot / UMR 1123 ECEVE-INSERM; 5 Université de Créteil; 6 Université de Caen / INSERM U1086 Anticipe; 7 Université de Strasbourg

**Keywords:** Social Sciences and Humanities, France, curriculum development, reflexivity, critical thinking, Social roles, ethics, public debate, patient participation, responsibilisation, biais, patient-centred care, personalized medicine, Philosophy, Anthropology, History, Arts, Litterature.

## Abstract

This article was migrated. The article was marked as recommended.

A public debate took place place in France in 2018 concerning ethical and social issues of biomedicine and life science and technologies. As faculty members of French medical schools and scholars in Social Sciences and Humanities, we contributed to introduce the central theme of health professionals education. What roles and what place should we assign to the social sciences and Humanities in preparing health professionals who will work in a transforming and largely unpredictable context? In this paper, we list 4 crucial issues for the present and the future of healthcare profession, concerning changes of medical roles; new biomedical concepts and innovations; long term consequences on health social contract; ethical issues in health care daily life settings. Then, we list 4 kinds of resources that are brought to students by Social Sciences and Humanities courses. They concern the connection to patients’s experiences the social and cultural construction of these experiences; the social responsibility of medical doctors; and the independence of their professional judgments. This is a plea for the development of reflexivity and critical thought backed up by well identified, well integrated and sufficiently developed Social Sciences and Humanities courses in French medical schools.

## Introduction

In today’s societies, the ethical, social and political challenges raised by the technical possibilities of the biomedicine, concerning artificial intelligence, the enhancement of human beings, procreation, etc, give rise to many passionate discussions. In many Western countries, diseases are more and more often chronic and related to environments, the population is ageing and inequalities in health care are growing. Health care system is affected by social and technological changes (mobile health, big data, robotics, etc) and is generating political challenges (increasing drug costs, development of preventive medicine, the biopolitics of risk, etc.). Patients are aware about their rights to solidarity and justice. They request health professionals to listen to their personal experiences, to be kept informed and participate in all decisions that concern them.

In 2018, the French National Consultative Ethics Committee for health and life sciences called for a public debate on this topic. This debate has been instrumental in the process of updating the regulatory and legal French framework concerning biomedical practices and research. However, while discussing the transformations that are affecting health care system and health professions, this public debate barely addressed educational issues. As teachers in medical schools and Social Sciences and Humanities scholars, we think that the training of health professionals is decisive to meet these challenges to society. It would be pointless to discuss social, technological and anthropological changes in care and medicine without considering how to best “equip” the players in the front line of change.

The following is a collective contribution by the “Collège des humanités médicales - Enseignants de sciences humaines et sociales en médecine et santé”. For us, the multidisciplinarity of the medical humanities, including history, anthropology, sociology, philosophy, ethics as well as the Arts and Literature is of crucial importance to the training of the future health professionals the population expects. Vis-a-vis social and ethical challenges and health policies, future health professionals need to develop certain resources and analytical capacities. They have to become aware of the presuppositions and the implications of their professional roles. They need to develop their imagination so as not to be locked into a narrow range of alternatives. Thus, what roles and what place should we assign to the social sciences and Humanities?

## How do we “equip” tomorrow’s health professionals?

The transdisciplinary field of medical humanities can contribute to the “equipment” of the future health professionals, in at least 4 fundamental dimensions.

(1) Medical students need to think about *their roles in the “medicine of the future”.* Today we are confronted with futuristic and even prophetic speeches which orientate our medical imagination. The job of “care coordinator” seems to be gaining ground over “caring for the body”. These words formulate ideals and standards which we assign to human life and reflect the central focus on health in consumer societies - the central focus on the body and its performances in social communications and the central focus on therapeutics in industrial societies ( Gaudillière, 2008). In the 20th century, the definition of health has become “the state of complete physical, mental and social wellbeing”, extending the concept of social medicine and public health invented in the 19th century. Starting in the 1970s, the Utopia of perfect health and the medicalisation of existence were denounced ( Illich, 1975). Precisely, far from the slogans vaunting or condemning these evolutions, social sciences and humanities document them with precision and in their historical depth. They make it possible for students to understand and assess the transformations of normalcy and the pathological in the light of scientific innovations, social values and public policies ( Foucault, 1973; Canguilhem, 1978, 2012). Today, from chronic diseases to the many pathologies occurring with age including cancers and depression, there is a vast multiplication in the number of interventions by many kinds of specialists (pharmacopoeias, rehabilitations, therapeutic education) for a wide range of motives (organic, psychological, cognitive, social, etc). Understanding at the same time the scientific and social constructions of health and diseases makes it possible to question the finalities and the sense of medical care and measure the observable or projected changes in the social functions of medicine.

(2) Health care students need *a critical analysis of the concepts of biomedicine.* For example, in the field of “personalised medicine”, the word “personal” concerns each individual, increasingly differentiated by molecular profile, membership of the most homogeneous possible sub-groups, with “layers”. The uniqueness on which “personalisation” is based is in fact the contrary to what is usually understood - the uniqueness of “people” following the philosophical steps forward in the 18 ^th^century and the promotion of autonomy and moral pluralism - which is what makes all of us singular and incommensurable (i.e. not able to be measured by the same standards). In “personalised” medicine, on the contrary, commensurability is a principle. As Xavier Guchet showed, each little difference is singularised as an element in a series. Insight from epistemology of medicine is essential when we come to question both the definition of diseases, therapeutic hopes and their ethical challenges ( Guchet 2016).

(3) Health care students need tools to understand *the long-term consequences of scientific and technical research.* For example, collecting health data shapes the infrastructure of tomorrow’s research and care. In the extension of health protocols already based on knowledge about individual risk factors, financing the health model and the mutualisation of risks will be transformed, with individualisation of risk and increased individual empowerment. Once the genetic and environmental data pertaining to an individual is ascertained, it is possible to identify all the “targets” for which a therapy exists or at least on which it is possible to intervene - for example, in nutritional genomics, prescribing food adapted to each specific genome. Everyone can then be obliged to make the “best choices for life” for their personal profile. Consequently, the bases of a solidarity model of Social Security are likely to be called into question. The rule of the “veil of ignorance” for individual choices of lifestyles and living conditions stated by Rawls (1972) appears to be more and more difficult to respect. Medical humanities visualise how changes in technical infrastructures can stress the social contract on which our health care systems are built.

(4) And of course social sciences and humanities are essential for *formulating reflections about ethics.* Health professionals are already questioning the assumption of responsibility for dependant people (autonomy, intimacy, sexuality, etc), care organisation and management, medical decisions for expensive treatments, the transformation of doctor/patient relationships with the new tools provided by mobile medicine, the availability of bodies for the donation of organs, etc. The health professionals we train today will inevitably be involved in controversies about artificial intelligence applications to health, interactions between bodies and technologies, manipulations of human beings, etc. They need to be given a thorough training in how to resolve conflicts in values and how to exert their profession in a context of moral pluralism and this to actively contribute to the forthcoming transformations in all aspects of health care. Social sciences and humanities sharpen students’ critical abilities by giving background to these transformations, highlighting the ruptures behind what on the surface appear to be seamless continuations or on the contrary underlining continuities in what is apparently a rupture. The Humanities, far from providing a common belief, augment ethical understanding, enable students to consider other viewpoints, restate problems, formulate them in words and consult other times and other cultures to throw a different light on them.

## What Social Sciences and Humanities can do

Medical humanities vector major transformations in training programmes. Here are four examples of what they can do to medical education:

(1) Thanks to their diversity and complementarity Medical Humanities contribute to *improving knowledge about private and social experiences and the biographical trajectories of each patient.* They throw new light on sharing sensitivities and seek to stem the erosion of empathy commonly observed during medical studies. They deconstruct the prejudices (of doctors and patients) and ensure that patients’ voices are not ignored nor overruled by the medical establishment. They contribute to truly individualised care, not adding incoherence, inconsistency and thoughtless conflicts to the physical, moral and social violence of every disease. Medical humanities contribute to democracy in health, deflate the presuppositions on which aristocratic, technocratic or scientistic medicine are predicated and promote partnership with patients in training, research and health care.

(2) Medical humanities provide the tools for *understanding the social and cultural constructs of health and disease -* the search for sense in universal phenomena like unhappiness, illness and death - what Francoise Héritier (1996) called the “thought stops”. Humans live through symbols which, depending on their social and cultural environments, they arrange and combine in their own ways. It is in this subtle balance between the universal and the specific that social sciences and the humanities can contribute to health care training. It is difficult to imagine medical practice that ignores the socio-cultural conditions of people’s existence, of the way they perceive life, their bodies, disease, death, the different cosmogonies in which they evolve and their concepts of health, care and treatment.

(3) Medical humanities contribute to the *critical reflexivity and responsibilisation of carers.* Over recent decades, standards and recommendations, economic constraints and productivity in health care dominate and eclipse the medical and ethical reasoning essential for responsible decision-making. Medical humanities help to clarify logic systems, values and interests in health care (including patients) and encourage everyone not to rush to conclusions and take an overview. In particular, they provide tools for discussions, shared decisions and the co-construction of medical protocols with patients. They also contribute to improving understanding the organisational, ethical and psychic factors of the suffering now evinced by carers and students. They aim to hit the “soft spots” to obtain global, well-considered diagnoses and imagine solutions together rather than to obtain “glossed-over” agreements.

(4) Medical humanities contribute to reinforcing *training in independence and judgement.* Today, in university hospitals, interns and externs often use Smartphone applications to find answers concerning diagnoses, molecules and care protocol recommendations. These applications give them quick and effective access to medical information. But the first problem concerns “solutionism” ( Morozov 2014) - a solution is suggested before the problem is completely formulated (miroring the multiple-choice questionnaires which dominate students’ assessments in French medical schools). The second problem is that these applications are sometimes (always?) financed by companies and may be biased. This is where judgement is essential. But it is so much more tiresome to open these “black boxes” than to use their recommendations. Medical humanities, specially Social Studies of Sciences and Technology provide resources to answer this acute challenge of judgemental training, along with methodology and critical reading training.

## Plea for the development of Humanities in medical schools

Our Collège pleads for the development of reflexivity and critical thought backed up by well identified, well integrated and sufficiently developed social sciences and humanities courses in medical training programmes ( Collège des enseignants de sciences humaines et sociales en médecine et santé, 2011). There is a fast-growing movement in medical humanities research and teaching innovations, testifying to the fact that they are increasingly recognized as essential components in the training of all care professionals, starting with doctors.

Social sciences and humanities help students understand the demographic, epistemic and technological transformations in today’s medicine better. They nourish reflections about carer/patient relationships as well as the medical context, policy issues and social factors in health care. They provide students with tools to think about and perfect the ways in which they work and their interactions with patients, their entourage and with all health professionals and society in general. These tools prepare them to enter their careers with an independent, responsible and patient-centred viewpoint and contribute to givingmeaning to their commitment to public health through many different, balanced and responsible choices of practice - an attitude well worth encouraging.

For this reason, we recommend the inclusion of social sciences and humanities in all vocational training courses in health care, to foster a new look in carer/patient relationships and to make students more aware of the new challenges of health care and prevention, reflexivity and critical thought. These concepts must be developed everywhere with help from social sciences and humanities specialists, in dialogue with clinicians and patients.

## Take Home Messages

• Medical education should be central in public debate about health, ethics and society.
• Future health professionals need to critically reflect on their changing roles, knowledge, social models and ethical equipment.
• Humanities and Social Sciences help to connect with and understand patients and offer necessary critical skills.
• To face present and future challenges for Medicine and Health, Medical schools should develop substantial and integrated programs in Social Sciences and Humanities.

## Notes On Contributors

All authors of this paper are members of the Executive Board of the College of Medical Humanities - Lecturers in Human and Social sciences in Medical and Health Schools in France ( http://colhum.hypotheses.org).

Nicolas Lechopier is assistant professor at the University of Lyon in philosophy, epistemology and ethics at the Faculté de Médecine Lyon Est. He is the president of the College of Medical Humanities. He currently develops research on patients participation, public health ethics and medical education.

Gilles Moutot is assistant professor in philosophy at the Faculty of Medicine of the University of Montpellier. His current research focuses on the tension between normativity and standardization in the field of medical practices and institutions in the spirit of Critical Theory. He wrote two books on Adorno.

Céline Lefève is assistant professor in philosophy at Sorbonne Paris Cité University. She is co-director of the Interdisciplinary Research Program, La Personne en médecine,and director of the Chair, Philosophy at Hospital (Assistance-Publique Hôpitaux de Paris/Ecole Normale Supérieure). She works on care ethics and medical education.

Maria Teixeira is assistant professor in medical anthropology. After spending years analyzing divination and therapeutic rites in West Africa, she works on biomedical medicine in Africa and in France. Currently, she is developing her reflection on the representations of the body and of the pain in people with sickle cell disease.

Roberto Poma is assistant professor in philosophy and history of medicine at the Université Paris-Est Créteil Val de Marne and develops research in historical epistemology and narrative medicine.

Guillaume Grandazzi holds a PhD in sociology and is associate professor at the University of Caen Normandy. He coordinates the “Espace de Reflexion Ethique de Normandie” and teaches ethics to medical students.

Anne Rasmussen is historian, professor of history of science at Strasbourg University and a member of the SAGE research unit (UMR 7363). She is interested in social and cultural history of biomedical sciences and healthcare, 19th-20th centuries. She is editor in chief of Le Mouvement social, a social history journal.

## References

[ref1] Canguilhem G. (1978) On the Normal and the Pathological. Dordrecht-Boston: D. Reidel Pub. Co. 10.1007/978-94-009-9853-7

[ref2] Canguilhem G. (2012) Diseases, Writings on Medicine. New York: Fordham University Press.

[ref3] Collège des enseignants de sciences humaines et sociales en médecine et santé (2011) Médecine, santé et sciences humaines, Manuel. Paris: Les Belles Lettres.

[ref4] Gaudillière J.-P. (2008) La médecine et les sciences, XIXe-XXe siècles. Paris: La Découverte.

[ref5] Illich I. (1975) Medical Nemesis. The Expropriation of Health. London: Calder & Boyars.

[ref6] Foucault M. (1973) The Birth of the Clinic: an Archaelogy of Medical Perception. New York: Pantheon Books.

[ref7] Guchet X. (2016) La Médecine personnalisée. Un essai philosophique. Paris: Les Belles Lettres.

[ref8] Héritier F. (1996) Masculin / féminin. La pensée de la différence. Paris: Editions Odile Jacob.

[ref9] Morozov E. (2014) To save everything, click here: technology, solutionism, and the urge to fix problems that don’t exist. London: Penguin Books.

[ref10] Rawls J. (1972) Theory of Justice. Oxford: Clarendon Press.

