# Supplementary material for: Health professionals prepared for the future. Why Social Sciences and Humanities teaching in Medical Faculties matter
Source: MedEdPublish (2016). 2018 Sep 5;7:195. [Version 1] doi: 10.15694/mep.2018.0000195.1 (PMC10701850; doi:10.15694/mep.2018.0000195.1)
Supplement: Peer review history

# Reviewer report: Reviewer response for version 1

**DOI:** 10.21956/mep.19665.r29358

**Published:** 2018-09-14

**Authors:** Alexandre Wenger

**Affiliations:** 1 Université de Genève

This review has been migrated. The reviewer awarded 4 stars out of 5 The article is clear and well organized. It proposes a rich general appraisal of the possible uses of medical humanities for health professionals, i.e. reflective and critical tools, which should enable a better adaptation to the future challenges of health systems.One of the strong points of the article is its discussion of the French situation. The authors of the article are all members of the French Collège des Humanités Médicales board with field experience in France. In the recent bioethical debates that have shaken France, the issue of medical education for future health professionals has been forgotten. The authors argue that the nature of teaching given to future physicians should be broached in public debates.Nevertheless, it is regrettable that the bibliography is almost exclusively French. The topics discussed have already been covered extensively in articles published in the leading English-language medical journals. The integration of an international dimension would also have made it possible to return specifically, at the end of the article, to the state of the debate in France. Given the quality of the whole, it would be interesting if the authors extended the scope of their text with one or two articles on the nature of the tools (what teaching aids, what course formats, etc.) and concrete solutions for the implementation of the humanities in a medical curriculum marked by a chronic lack of time.

Reviewer Expertise:

NA

No decision status is available

# Reviewer report: Reviewer response for version 1

**DOI:** 10.21956/mep.19665.r29356

**Published:** 2018-09-06

**Authors:** Alexandre Berkesse

**Affiliations:** 1 Université de Montréal

This review has been migrated. The reviewer awarded 4 stars out of 5 This article is a pedagogical synthesis of questions that we can no longer avoid with the challenges that come in a short-term perspective for the healthcare ecosystem and its actors (considering that the patients are the main actors of care on a daily basis, that is to say almost all citizens).Social sciences and humanities are mostly denigrated in the biomedical paradigm (based on positivism and focused on a self-referential efficiency rather than on a contextual and relational relevance), that’s why the questions raised in this article needs to be brought to the attention of the influencial players in medical education.I would advise the authors to specify the contextual details in the first paragraph (too generic, this does not illustrate their fine mastery of the subject or the added value of SSH to understand those issues) and to play the game of the actual epsitemological forces in place (ex: patient partnership is one of the most efficient way to train competent physicians or to decrease the dependance of patients to the healhcare system rather than to present it only as a democratic necessity or a moral injonction).A short comment could be added to highlight the flip side of the proposition : in a pedagogical and a clinical context which crushes the complexity and diversity of perspectives, to equip students to open their eyes to the contradictory injunctions to which they are subjected can also lead to increase their anxiety, their feeling of helplessness rather than their emancipation. It is a risk to take but it must be said.Also, the social contract part would gain in strength if the role of the State and the citizens would be integrated in the balance of power that constitutes it and makes it evolve.Finally, the focus on education of the future healthcare professionnals which they sensitize us is not a luxury but a necessity. This article is a necessity.

Reviewer Expertise:

NA

No decision status is available

# Reviewer report: Reviewer response for version 1

**DOI:** 10.21956/mep.19665.r29357

**Published:** 2018-09-05

**Authors:** Trevor Gibbs

**Affiliations:** 1 AMEE

This review has been migrated. The reviewer awarded 4 stars out of 5 A very well written paper, well reference and well structured, giving a sound argument for the inclusion of the social sciences and the humanities withing the undergraduate medical curriculum. I learned a lot from this paper and found it difficult to argue against such sound proposals, backed by solid reasoning. I like the structure of the paper which encouraged a logical flow to the authors' specific flow. My only addition to how the paper might have been improved would be by the inclusion of some specific examples in the final section- the how to bit of the argument.I think this paper should be read by all involved in curriculum development and perhaps by those medical students about to embark on their studies.

Reviewer Expertise:

NA

No decision status is available
